# Semi-automated MicroED system unveils multiple polymorphs in fish-derived guanine crystals

**DOI:** 10.1107/S2053229626006236

**Published:** 2026-06-22

**Authors:** Minato Nakazawa, Seishu Hayashi, Keita Tanaka, Kenji Iwasaki, Makoto Goda, Yusuke Yamada, Naruhiko Adachi

**Affiliations:** a Life Science Center for Survival Dynamics, Tsukuba Advanced Research Alliance (TARA), University of Tsukuba, 1-1-1 Tennodai, Tsukuba, Ibaraki 305-8577, Japan; b Institute of Photonics Medicine, Hamamatsu University School of Medicine, 1-20-1 Handayama, Chuo-ku, Hamamatsu, Shizuoka 431-3192, Japan; c International Center for Synchrotron Radiation Innovation Smart (SRIS), Tohoku University, 468-1 Aramaki aza aoba, Aoba-ku, Sendai, Miyagi 980-0845, Japan; University of North Texas at Dallas, USA

**Keywords:** crystal structure, MicroED, semi-automated system, biogenic guanine crystals, cryo-EM, polymorphism

## Abstract

Certain fish display silvery or blue colour, not through pigments, but through specialized guanine crystals that exist within iridophore cells in their skin. Within these cells, guanine (C_5_H_5_N_5_O) crystals are precisely arranged in stratified layers that reflect incident light, resulting in structural colour. This phenomenon has garnered growing scientific attention due to its potential applications in advanced materials. However, the minute size of guanine crystals significantly hinders their structural characterization. Guanine crystals exist in multiple polymorphic forms. Previous powder X-ray dif­frac­tion (PXRD) studies have revealed that fish-derived guanine primarily adopts the anhydrous α- and/or β-polymorphs. More recently, MicroED analyses have identified the β-polymorph in salmon. Nonetheless, the crystal forms in other fish species remain insufficiently characterized. Due to their nanoscale dimensions and sensitivity to electron irradiation, reliable structural determination requires data col­lection from a large number of crystals. Here we established a semi-automated MicroED data col­lection and processing system. Initial validation was performed using synthetic guanine crystallized under acidic and basic conditions. Subsequently, guanine crystals were analyzed from three fish species: Pacific saury, Pacific cutlassfish and blue damselfish. Our findings revealed the presence of both α- and β-polymorphs in all three species, corroborating previous PXRD reports and demonstrating the efficacy of our system for the structural investigation of submicrometer-scale biogenic crystals.

## Abstract 2

A semi-automated MicroED data col­lection and processing system was established and shown to be suitable for the structural investigation of submicrometer-scale biogenic crystals.

## Introduction

Certain fish species exhibit silvery or blue colour, not through pigmentation, but through biogenic crystalline architectures. These fish possess specialized iridophore cells within their skin, where guanine crystals are precisely organized into layered structures within the cytoplasm. This multilayered configuration produces structural colour through optical inter­ference, that is, through the inter­action of visible light with microscopically ordered structures rather than through light absorption by pigments. In silvery fish, the cytoplasm spacings between crystal layers varies over a broad range, leading to broadband reflection, in which most or all wavelengths of light are reflected (Gur *et al.*, 2017 ▸). Conversely, in blue fish, the cytoplasm spacings between crystal layers is uniform, producing narrowband reflection, in which only specific wavelengths of light are selectively reflected (Gur *et al.*, 2017 ▸). From the perspectives of biomimetics and biomineralization, fish structural colour has garnered significant research inter­est due to its potential applications in cosmetics, coatings and advanced materials science (Sano *et al.*, 2016 ▸; Luo *et al.*, 2019 ▸; Chen *et al.*, 2021 ▸). Consequently, the precise crystal structure of guanine within iridophore cells – fundamental to the generation of structural colour – is a subject of considerable scientific intrigue. However, guanine crystals derived from fish are inherently minute, typically at the submicrometer scale, posing substantial challenges for structural determination.

A longstanding question concerns the polymorphic diversity of guanine crystals in fish. Guanine is known to crystallize in multiple structural forms, including guanine monohydrate, the α-, β- and γ-polymorphs of anhydrous guanine, and guanine sodium salt crystals (Gur *et al.*, 2016 ▸). The crystal structure of anhydrous synthetic guanine was first determined in 2006 using synchrotron X-ray analysis (Guille *et al.*, 2006 ▸). The powder X-ray dif­frac­tion (PXRD) pattern of carp-derived crystals exhibited a resemblance to the theoretical PXRD pattern of anhydrous synthetic guanine, initially leading to the assumption that fish-derived guanine adopts the same polymorphic form (Levy-Lior *et al.*, 2008 ▸). However, subsequent PXRD analyses suggested that guanine crystals in carp, salmon and sea bass more likely correspond to an alternative polymorphic form (Hirsch *et al.*, 2015 ▸; Pinsk *et al.*, 2022 ▸). The former was classified as the α-polymorph and the latter as the β-polymorph, yet no conclusive criterion has been established to unambiguously assign the polymorphic form of fish-derived crystals. Under these circumstances, electron dif­frac­tion has become a powerful technique for determining the structures of submicrometer-scale crystals (Dorset, 1996 ▸). Electron dif­frac­tion was later applied to ultra-thin protein crystals, an approach that became widely known as MicroED (Shi *et al.*, 2013 ▸) or 3D ED (Yonekura *et al.*, 2015 ▸), and was subsequently extended to small-mol­ecule crystallography at the submicrometer scale (Jones *et al.*, 2018 ▸; Yonekura *et al.*, 2023 ▸; Unge *et al.*, 2025 ▸). More recently, MicroED ex­peri­ments indicated that guanine crystals isolated from salmon correspond to the anhydrous β-polymorph (Wagner *et al.*, 2024 ▸).

Despite these advances, the polymorphic characteristics of guanine crystals in fish species other than salmon, carp and sea bass remain largely uncharacterized. It remains uncertain whether taxonomic groups outside *Salmoniformes* – such as Pacific saury (*Beloniformes*) and Pacific cutlassfish (*Perciformes*) – exclusively adopt the anhydrous β-polymorph. Given the considerable evolutionary divergence among these taxonomic groups, it is plausible that different species employ distinct guanine polymorphs. Moreover, while previous studies have examined the guanine crystal structures of fish with constant structural colour (*e.g.* carp, salmon and sea bass), the polymorphic com­position of species capable of dynamic colour modulation – such as the blue damselfish, neon tetra and zebrafish – remains unexplored. At the cellular level, the mechanisms governing colour modulation are well established: these species dynamically regulate their structural colouration by rearranging guanine crystal layers within their iridophores (Gur *et al.*, 2024 ▸). However, the mol­ecular mechanisms underlying this process remain elusive, partly because the crystal structures and polymorphic forms of these guanine crystals have not been fully characterized.

Addressing these fundamental questions presents several technical challenges. The first pertains to sample throughput: a routine and efficient MicroED data col­lection and processing pipeline must be established to enable structural analysis of guanine crystals across a diverse range of fish species. Several automated and semi-automated workflows for MicroED data col­lection and processing have pre­viously been reported (Cichocka *et al.*, 2018 ▸; Ge *et al.*, 2021 ▸). The second challenge concerns crystal size. While guanine crystals derived from salmon are relatively large (several µm × 1 µm × 25 nm) (Wagner *et al.*, 2024 ▸), those from blue damselfish are significantly smaller (∼0.5 µm × 0.2 µm × 10–20 nm), rendering them more difficult to isolate and highly susceptible to electron-beam-induced damage. For such minute crystals, dif­frac­tion data must be collected from a large number of individual crystals and merged to ensure high com­pleteness. The third challenge involves structural heterogeneity: fish species beyond salmon may harbour multiple guanine polymorphs, some of which may exist in minor proportions (Jordan *et al.*, 2012 ▸). Overcoming these obstacles may require the screening, acquisition, processing and merging of dif­frac­tion data from hundreds to thousands of individual submicrometer-sized crystals to achieve reliable structure determination in challenging biogenic samples. Together, these con­sid­er­ations make automating the MicroED data-col­lection and processing workflow highly advantageous.

In this study, we developed a semi-automated MicroED data col­lection and processing system based on the CRYO ARM200, Rio 16M and *SerialEM* configuration. The system was initially validated using synthetic guanine crystallized under several conditions. Diffraction data were collected from 143 crystals obtained under acidic conditions, corresponding to the monohydrate form, and from 216 crystals obtained under basic conditions, corresponding to the anhydrous α- and β-polymorphs. Subsequently, structural analyses of 506 guanine crystals from Pacific saury, 651 from Pacific cutlassfish and 2127 from blue damselfish revealed the presence of the anhydrous β-polymorph, as well as the anhydrous α-polymorph. These findings are con­sis­tent with prior PXRD ob­ser­va­tions. Our results demonstrate the efficacy of our semi-automated MicroED pipeline in resolving the structural heterogeneity of submicrometer-scale crystals in biological systems.

## Materials and methods

### Crystallization of synthetic guanine

The crystals of synthetic guanine were prepared according to a previous study (Gur *et al.*, 2016 ▸). In brief, guanine solutions at concentrations of 1 mg ml^−1^ were prepared in 1 *M* HCl or 1 *M* NaOH. The pH was adjusted to pH 2 or 10 using 1 *M* NaOH or 1 *M* HCl, respectively. The solutions were left undisturbed for several days at room tem­per­a­ture until crystals formed. The resulting crystal suspension was filtered to collect the crystals, which were then transferred onto a glass slide and allowed to dry com­pletely.

### Sample preparation of fish-derived guanine crystals

To prepare guanine crystals from fish, Pacific saury (saury) and Pacific cutlassfish (cutlassfish) were procured from a local fish market, while blue damselfish (bluefish) were obtained from an online supplier (Aqua Marine Fujimi). Experiments involving fish were conducted in accordance with the Regulations on Animal Experiments at the University of Tsukuba. Commercially available fish were used and only the minimum amount of tissue required for crystal extraction was collected. Fish skins were separated from the underlying muscle tissue and scales were carefully removed using tweezers. The fish skins were then immersed in physiological saline solution (154 m*M* NaCl). Any remaining scales and residual muscle tissue were subsequently removed, after which the skins were transferred to fresh physiological saline solution.

The prepared fish skins were subsequently homogenized using a Dounce homogenizer. A 2 ml aliquot of DNase I solution [0.02% DNase I (Worthington) in 130.7 m*M* NaCl, 2.7 m*M* KCl, 5.6 m*M*D-(+)-glucose, 5 m*M* Tris and 0.2 m*M* EDTA] was added and the mixture was homogenized under moderate conditions. Another 2 ml of DNase I solution was then added, followed by incubation at 37 °C for 30 min. The resulting suspension was filtered through a cell strainer and the flow-through was collected. Subsequently, 1 ml of trypsin–EDTA solution (SIGMA) was added and the mixture was incubated at 37 °C for 60 min. The solution was filtered again and the flow-through was retained. Next, 1 ml of DNase I solution and 1 ml of trypsin solution were added, followed by centrifugation at 200 × *g* at room tem­per­a­ture (RT) for 15 min. The supernatant was collected, 2 ml of CMF-Ringer buffer [130.7 m*M* NaCl, 2.7 m*M* KCl, 5.6 m*M*D-(+)-glucose, 5 m*M* Tris and 0.2 m*M* EDTA] was added and the mixture was then centrifuged under the same conditions. The supernatant was retained for subsequent preparation.

Guanine crystals were isolated using a sucrose gradient, which was prepared by sequentially layering, from bottom to top, a saturated sucrose solution (5.84 *M*), a two-thirds saturated sucrose solution (3.89 *M*) and the pre­viously collected supernatant. The layered gradient was centrifuged at 1000 × *g* at RT for 20 h. A distinct white band appearing in the upper third of the tube, which con­tained guanine crystals, was carefully retrieved. The recovered sample (500 µl) was mixed with an equal volume of MilliQ water and centrifuged at 5000 × *g* at RT for 4 h. The supernatant was discarded and 1 ml of MilliQ water was added, followed by gentle tapping and centrifugation at 5000 × *g* at RT for 15 h. The supernatant was removed, and the process was repeated with 0.5 ml of MilliQ water, followed by centrifugation at 4000 × *g* at RT for 20 h. The supernatant was removed and the resultant white pellets corresponded to purified guanine crystals. For final preparation, because guanine crystals are generally considered insoluble in pure water, 20–50 µl of MilliQ water was added and the sample was gently resuspended and briefly centrifuged. The resulting crystal-con­taining suspension was utilized for grid preparation.

### Grid preparation and data col­lection for MicroED

For grid preparation, holey carbon grids (Qu­anti­foil Cu300, R1.2/1.3) were used. The grids were rendered hydro­philic by glow discharge for 30 s in a vacuum (11 mA) using a PIB-10 instrument (Vacuum Device) before use. For synthetic crystals, the grid was flipped over and placed directly on top of the crystals to allow crystal attachment. For the fish-derived crystals, 3 µl of the crystal suspension was applied to the grid and excess liquid was removed by blotting from the backside of the grid with filter paper. The grids were then left to dry com­pletely before being transferred to the cryogenic electron microscope.

Diffraction data were acquired on a CRYO ARM200 (JEM-Z200FSC) microscope operated at 200 kV with a Rio 16M detector (Gatan). Automated data col­lection was conducted using *SerialEM* (Mastronarde, 2005 ▸) with a custom script originally developed by Drs Makino, Yanagisawa and Nakane, and later modified at the University of Tsukuba and Tohoku University. The illumination settings were as follows: emission, 1.4–2 µA; A2, 7.37–7.43 kV; spot, 7; angle, 6; magnification, ×30000; condenser lens aperture (CLA), 70 µm; beam di­ameter, 2 µm; parallel illumination; and flux, 0.05 e^−^ Å^−2^ s^−1^. For dif­frac­tion data col­lection, the microscope was switched to dif­frac­tion mode and data were recorded at 2k × 2k with continuous rotation at 1° s^−1^ and a frame rate of 1 frame/s. For screening sessions, approximately ten crystals were selected and dif­frac­tion data were collected over a tilt range of ±30°. This angular range was sufficient to determine preliminary unit-cell parameters and space-group information, and to assess whether the sample was suitable for large-scale data col­lection. For overnight data col­lection, approximately 200 newly selected crystals were measured over a tilt range of ±65°. This angular range was chosen because ±70° represents the mechanical tilt limit of the CRYO ARM 200 microscope and a small margin is required for stable operation. Because the fish-derived guanine crystals investigated in this study had not been characterized pre­viously by MicroED, we adopted a conservative low-dose data col­lection strategy using an electron flux of approximately 0.05 e^−^ Å^−2^ s^−1^ for 130 s, corresponding to a total exposure of approximately 6.5 e^−^ Å^−2^.

For low-mol­ecular-weight samples, nominal camera lengths of 400 (calibrated camera length, 408 mm; edge, 0.55 Å), 500 (calibrated camera length, 509 mm; edge, 0.69 Å) and 600 mm (calibrated camera length, 609 mm; edge, 0.83 Å) were often used. Camera length calibration was performed using the dif­frac­tion ring pattern from an evaporated aluminium grid (Alliance Biosystems). Representative dif­frac­tion images of the synthetic and fish-derived crystals are shown in Fig. S1 (see supporting information). Under the default settings of the Rio detector, certain pixels exhibit negative values in beam-blank condition images. Because *SerialEM* truncates negative values in Rio data, weak signals may be removed. To mitigate this artifact, the dark reference was modified by incorporating the gain reference using Modify_dark_ref.s. As a result, the histogram of pixel counts in beam-blank images exhibited an average of 100 with a standard deviation of 16.

### Data analysis for MicroED

During data col­lection, the dif­frac­tion data were stored on a 250 TB network-attached storage system and automatically processed by *DIALS* (Beilsten-Edmands *et al.*, 2020 ▸; Vyprit­skaia *et al.*, 2025 ▸) up to the scaling step. This process was exe­cuted using custom scripts, including monitor_data­set.sh, process_auto.sh and filter_blank.py (https://github.com/GKLabIPR/MicroED), originally developed by Drs Yamashita and Nakane, and further modified at the University of Tsukuba and Tohoku University. On the basis of the output, users could decide whether to move on to the next sample or proceed with additional data col­lection. At this stage, several distinct sets of unit-cell parameters were typically obtained. Some corresponded to the target guanine crystals, including potential polymorphs, whereas others could arise from ice contamination, multiple overlapping crystals or incorrectly indexed data­sets.

To determine the appropriate space group(s), data­sets exhibiting the predominant set of unit-cell parameters were manually selected and merged using a custom script (merge.sh). After specific unit-cell parameters were obtained, collected data­sets could be reprocessed with the specified unit-cell parameters as arguments for dials.index. In this study, the following unit-cell parameters (Å, °) were used: (3.6, 11, 16.5, 90, 96, 90) for synthetic guanine pH 2 (synG); (3.6, 8.8, 18.5, 90, 90, 83) for the first type of synthetic guanine pH 10 (synGβ) and fish-derived crystals (sauryGβ, cutlassfishGβ and bluefishGβ); and (3.6, 9.8, 16.5, 90, 96, 90) for the second type of synthetic guanine pH 10 (synGα) and fish-derived crystals (sauryGα, cutlassfishGα and bluefishGα).

From the reprocessed data­sets, the highest-resolution data­sets were selected and merged as follows: 14 for synG; 9 for synGβ; 13 for synGα; 23 for sauryGβ; 37 for sauryGα; 8 for cutlassfishGβ; 23 for cutlassfishGα; 9 for bluefishGβ; and 4 for bluefishGα. In this study, the resolution of each data­set was defined as the highest-resolution shell for which CC_1/2_ remained greater than 0.3 during *DIALS* processing, whereas the resolution of the merged data­set was defined as the highest-resolution shell for which CC_1/2_ remained greater than 0.5. Initial structures were solved using *SHELXT* (Sheldrick, 2008 ▸; Sheldrick, 2015*a* ▸) and further refined using *SHELXL* (Sheldrick, 2008 ▸; Sheldrick, 2015*b* ▸). All structure refinements were performed using the Peng-1999 (4G) electron scattering factors (Peng, 1999 ▸). All non-H atoms were located directly during structure solution using *SHELXT*. Atom types were assigned based on the known chemical structure of guanine. H atoms were identified from Fourier difference maps and included in the refinement. Initially, all atoms, including H atoms, were refined freely without geometrical restraints or constraints. An exception was the water mol­ecule in synG, for which one H atom could not be refined to a chemically reasonable position and occupancy. This ob­ser­va­tion is con­sis­tent with the H-atom disorder pre­viously reported for guanine monohydrate (Thewalt *et al.*, 1971 ▸). For the final refinement, H atoms were placed in riding positions to maintain chemically reasonable N—H bond lengths in electron-dif­frac­tion refinements. To partially com­pensate for dynamical scattering effects in electron dif­frac­tion, an extinction parameter was refined during the final refinement cycles. In this context, the extinction parameter should not be inter­preted as a physically rigorous description of extinction in the con­ven­tional X-ray crystallographic sense. Rather, it serves as an empirical correction that partially accounts for systematic intensity deviations arising from dynamical scattering. Structure calculations were performed through the *OLEX2* GUI (Dolomanov *et al.*, 2009 ▸). Data col­lection and refinement statistics of the synthetic and fish-derived guanine crystals are summarized in Tables S1 and S2, respectively, in the supporting information. Structural com­parisons and visualizations were performed using *Mercury* (Macrae *et al.*, 2020 ▸).

## Results

### Development of a semi-automated system for MicroED data col­lection and processing

**Figure 1:**
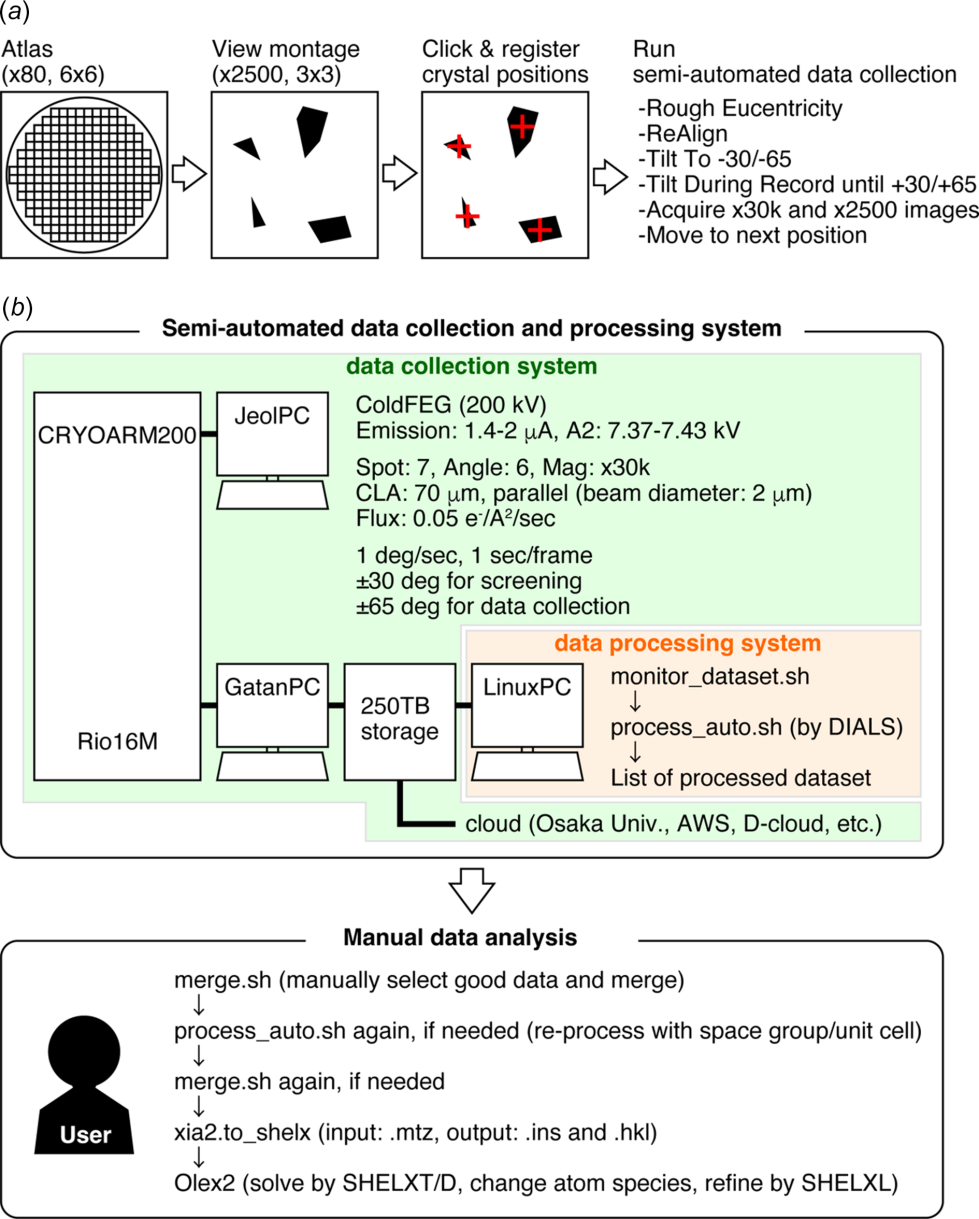
Semi-automated system for MicroED data col­lection and processing. (*a*) Overall workflow of MicroED data col­lection in this study. (*b*) Schematic diagram of the semi-automated system for MicroED data col­lection and processing. Structure determination was performed manually.

To enhance the efficiency and throughput of MicroED ex­peri­ments, we established a collaborative online research network connecting the University of Tokyo, KEK, Osaka University, the University of Tsukuba and Tohoku University. This inter­disciplinary coordination – particularly among microscopists, crystallographers and beamline scientists – enabled the successful implementation of a semi-automated MicroED data col­lection and processing system at the University of Tsukuba. The overall workflow for MicroED data col­lection is outlined in Fig. 1 ▸(*a*). In brief, the procedure begins with the acquisition of a low-magnification overview of the entire grid as an Atlas (nominal magnification: ×80), followed by higher-magnification imaging at the Square level (nominal magnification: ×2500). Crystal positions are then registered and semi-automated data col­lection is initiated. Compared with commercially available software, a key advantage of our system lies in the process of crystal position registration. Conventional software requires physical stage movement to each crystal position for registration, a time-intensive procedure requiring approximately 2 h for 100 crystals (Tsunekawa *et al.*, 2023 ▸; Koga *et al.*, 2024 ▸). In contrast, our system allows direct crystal position registration from square images *via* a simple point-and-click inter­face, reducing the registration time to only 10 min for 100 crystals. This workflow, originally developed at the University of Tokyo and Osaka University, was executed using a CRYO ARM200 electron microscope equipped with a Rio detector and operated *viaSerialEM* software [green shading in Fig. 1 ▸(*b*)]. During the screening session, the stage was tilted within a ±30° range to optimize time efficiency. For data col­lection, the stage was tilted within ±65°, constrained by the 70° tilt limit of the CRYO ARM200.

The acquired data­sets were automatically transferred to a dedicated data-processing pipeline [indicated with orange shading in Fig. 1 ▸(*b*)]. Data reduction and processing were carried out using the *DIALS* software suite, with automation facilitated by custom scripts (monitor_data­set.sh, process_auto.sh, filter_blank.py and merge.sh). This workflow, initially developed at Osaka University, automatically generated output in a text file, including sample ID, resolution, space group and unit-cell parameters. Additionally, an HTML-based summary table was produced, displaying sample ID, crystal images, dif­frac­tion images at 0°, resolution, space group and unit-cell parameters. At this stage, users could assess whether to proceed with the next sample or conduct additional data col­lection at ±65°.

### MicroED analysis of synthetic guanine crystals

**Figure 2:**
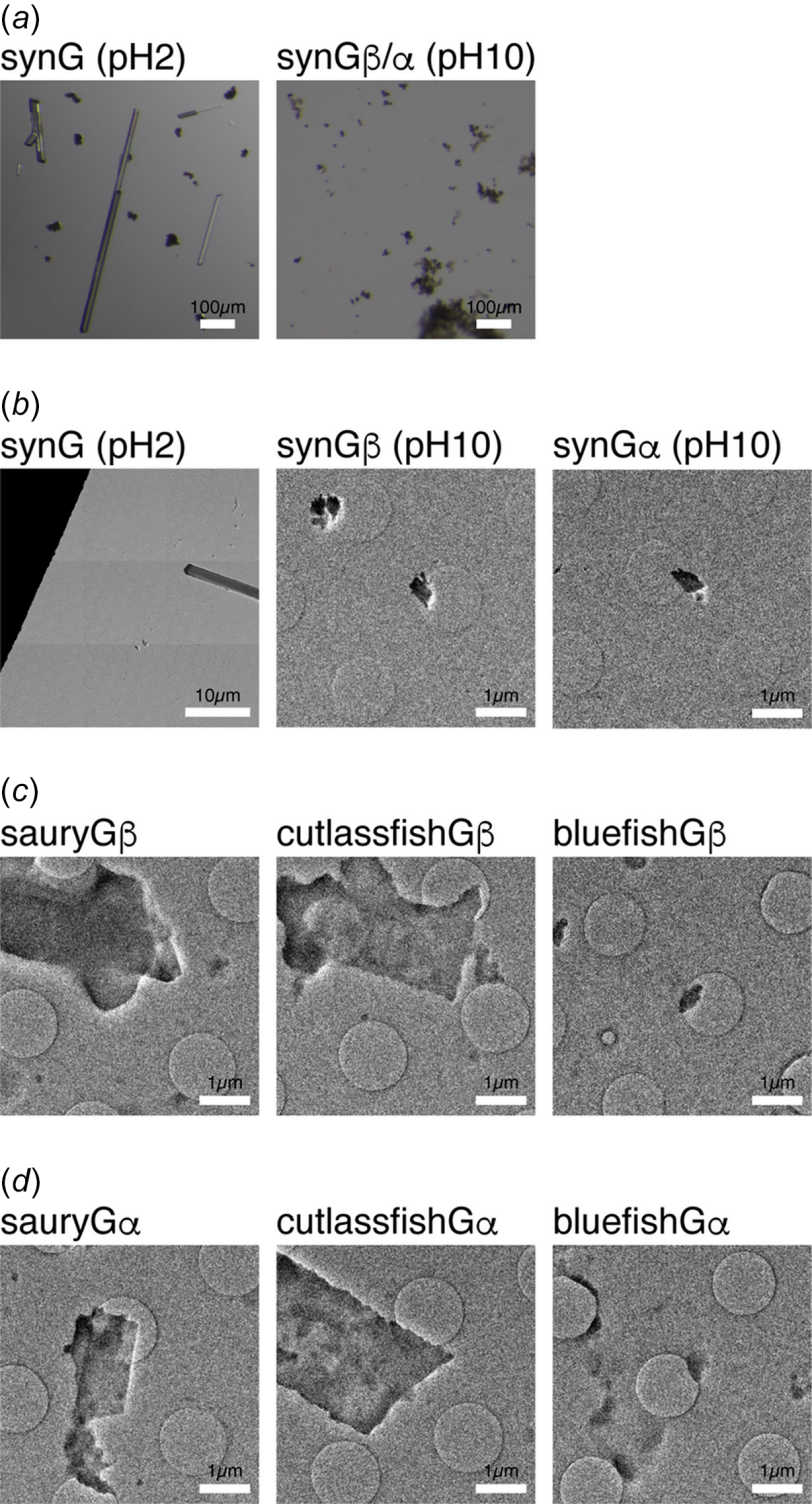
Representative images of guanine crystals. (*a*) Light microscope images of synG (pH2) and synGβ/α (pH10) crystals. (*b*) Cryo-EM images of synG, synGβ and synGα crystals. (*c*) Cryo-EM images of fish-derived Gβ crystals. (*d*) Cryo-EM images of fish-derived Gα crystals.

To validate the performance and reliability of the developed MicroED system, we first conducted a structural analysis of synthetic guanine crystals under controlled pH conditions. Under acidic conditions (pH 0–3), guanine is known to crystallize as a monohydrate, whereas under basic conditions (pH 7–13), it adopts the anhydrous α- and β-polymorphs (Gur *et al.*, 2016 ▸). Synthetic guanine was crystallized at pH 2 (acidic) and 10 (basic), yielding rod-like crystals exceeding 10 µm in length and small particulate crystals measuring less than 1 µm, respectively. Synthetic guanine crystals were observed by light microscopy [Fig. 2 ▸(*a*)] and cryo-EM [Fig. 2 ▸(*b*)]. For the pH 2 and 10 conditions, 143 and 216 data­sets were collected, respectively (Tables 1 ▸ and S1). Among these, 48 and 35 data­sets were processed successfully through the automated pipeline up to the scaling step. The unit-cell parameters determined for the acidic condition corresponded to synG (3.6, 11, 16.5, 90, 96, 90), con­sis­tent with the known parameters of guanine monohydrate. Under basic conditions, two distinct sets of unit-cell parameters were identified: synGβ (3.6, 8.8, 18.5, 90, 90, 83) and synGα (3.6, 9.8, 16.5, 90, 96, 90).

Reprocessing with predefined unit-cell parameters of synG, synGβ and synGα resulted in 16, 9 and 19 data­sets, respectively. Among these, the highest-resolution data­sets (14, 9 and 13, respectively) were selected for final merging. Our system successfully achieved 100% com­pleteness and resolutions of 0.56, 0.58 and 0.55 Å, respectively. The initial structures, assigned to space groups *P*2_1_/*n*, *P*2_1_/*n* and *P*2_1_/*c* were solved using *SHELXT* and subsequently refined with *SHELXL*, yielding *R*_1_ values of 16.00, 14.15 and 12.04%, respectively. For the crystals with the unit-cell parameters of synG and synGα, the results were con­sis­tent with pre­viously reported structures of guanine monohydrate (Thewalt *et al.*, 1971 ▸) and synGα (Guille *et al.*, 2006 ▸), respectively (orange and blue coloured data in Table 1 ▸, respectively). For the crystals with the unit-cell parameters of synGβ, a lattice transformation from *P*2_1_/*n* to *P*2_1_/*c* was performed to facilitate direct com­parison with the unit-cell parameters of the anhydrous β-polymorph of guanine, following a previous study (Wagner *et al.*, 2024 ▸). This transformation confirmed structural consistency with pre­viously reported synGβ (green coloured data in Table 1 ▸). To the best of our knowledge, this study provides the first single-crystal structure determination of synGβ. Previous structural information for this polymorph was primarily derived from powder X-ray dif­frac­tion data (Wagner *et al.*, 2024 ▸).

**Figure 3:**
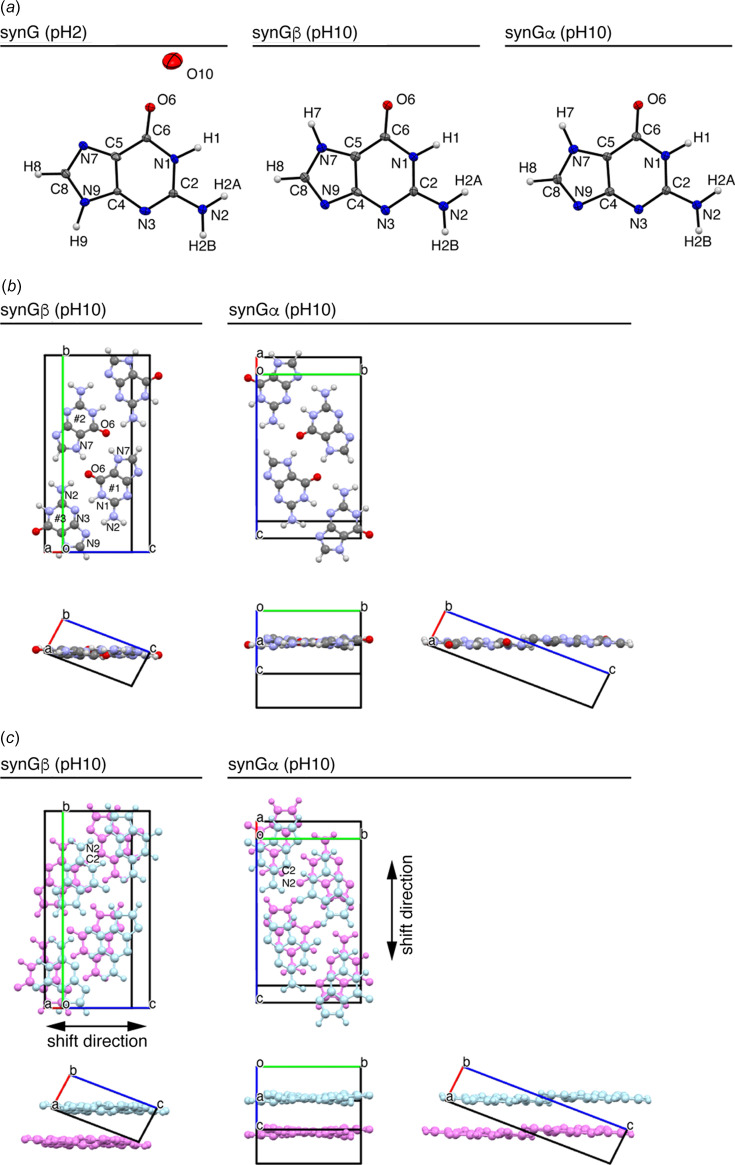
(*a*) Crystal structures of synG, synGβ and synGα determined from MicroED data. (*b*) Crystal packing of synGβ and synGα determined from MicroED data. (*c*) Direction of displacement of the stacked planar sheets of guanine in synGβ and synGα crystals.

While guanine mol­ecules exist as keto-N9H tautomers in synG crystals, they exist as keto-N7H tautomers in synGα and synGβ crystals, as reported pre­viously [Fig. 3 ▸(*a*)]. To verify the tautomeric assignments, the guanine structures were re-refined after removal of all H atoms, and the corresponding *F*_o_–*F*_c_ difference maps were calculated. The omitted H atoms were clearly visible as positive peaks in the difference maps (Fig. S2). As in the previous report, synGα and synGβ share an essentially identical hy­dro­gen-bonded layer, which is nearly planar (Hirsch *et al.*, 2015 ▸) [Fig. 3 ▸(*b*)]. The O6_1_/N7_1_ side of guanine #1 faces the O6_2_/N7_2_ side of guanine #2, forming two hy­dro­gen bonds: O6_1_⋯H7_2_—N7_2_ and N7_1_—H7_1_⋯O6_2_. Similarly, the O6_1_/N1_1_/N2_1_ side of guanine #1 faces the N2_3_/N3_3_/N9_3_ side of guanine #3, forming three hy­dro­gen bonds: O6_1_⋯H2*B*_3_—N2_3_, N1_1_—H1_1_⋯N3_3_ and N2_1_—H2*A*_1_⋯N9_3_. The primary distinction between synGα and synGβ lies in the direction of displacement of the stacked planar sheets within the hy­dro­gen-bonded layer [Fig. 3 ▸(*c*)]. In the synGα, the hy­dro­gen-bonded layers are offset along the N2—C2 axis of the guanine mol­ecules, whereas in the synGβ, they are offset in the direction perpendicular to the N2—C2 axis of the guanine mol­ecules. Collectively, these results are con­sis­tent with previous studies and underscore the reliability and accuracy of our semi-automated MicroED workflow.

### MicroED analysis reveals two distinct unit-cell parameters in fish-derived crystals

To investigate the polymorphic characteristics of guanine crystals in fish species beyond salmon, guanine crystals were extracted from the skin of saury (Beloniformes), cutlassfish (Perciformes) and bluefish (Perciformes), and subsequently applied to a TEM grid. Crystal size varied among species: saury and cutlassfish exhibited crystal dimensions of approximately several µm × 1–2 µm, whereas those from bluefish were significantly smaller, measuring less than 1 µm [Figs. 2 ▸(*c*) and 2(*d*)]. A total of 506, 651 and 2127 data­sets were collected for saury, cutlassfish and bluefish, respectively (Table 2 ▸ and S2). Among these, 63, 34 and 21 data­sets, respectively, were successfully processed through the automated pipeline up to the scaling step. In all cases, two distinct sets of unit-cell parameters, corresponding to Gβ and Gα, were identified, indicating the presence of multiple guanine polymorphs across these species.

### MicroED analysis of fish-derived Gβ crystals

**Figure 4:**
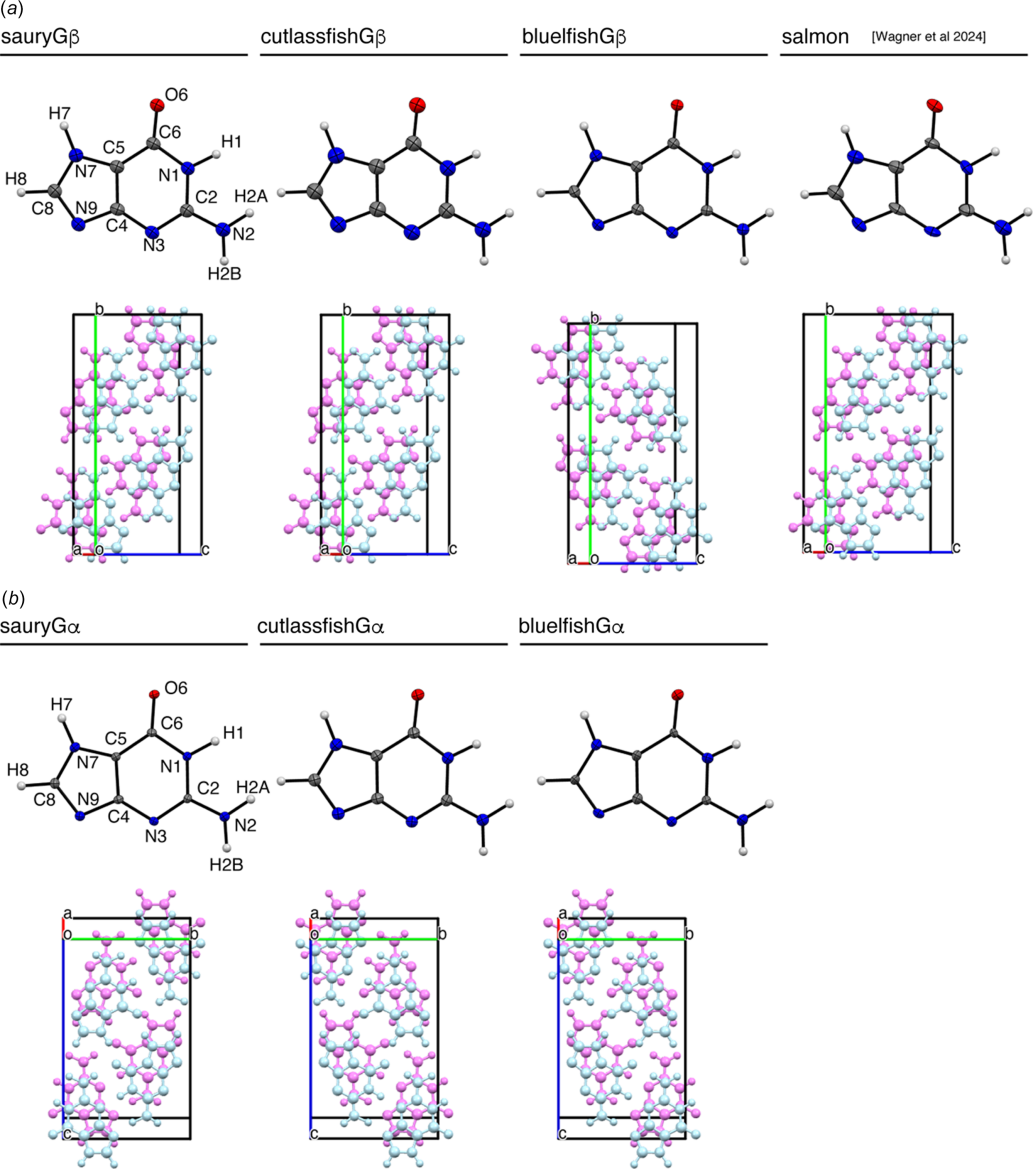
(*a*) Crystal structure and packing of fish-derived Gβ determined from MicroED data. (*b*) Crystal structure and packing of fish-derived Gα determined from MicroED data.

For the fish-derived Gβ crystals (sauryGβ, cutlassfishGβ and bluefishGβ), reprocessing with a predefined unit cell of (3.6, 8.8, 18.5, 90, 90, 83) resulted in 29, 9 and 14 data­sets, respectively. From these, the 23, 8 and 9 highest-resolution data­sets were selected for final merging (green coloured data in Table 2 ▸). As pre­viously reported, fish-derived guanine crystals exhibit a plate-like morphology, leading to preferred orientation on the TEM grid. Furthermore, their small size and high susceptibility to electron-beam-induced damage pose considerable challenges for high-resolution data acquisition. However, by implementing a high-throughput data col­lection strategy followed by a best-selection approach, we achieved a com­pleteness exceeding 85% and resolutions of 0.55, 0.57 and 0.57 Å, enabling successful structure determination. The initial structure, assigned to space group *P*2_1_/*n*, was solved using *SHELXT* and subsequently refined with *SHELXL*, yielding *R*_1_ values of 9.46, 9.44 and 7.78% for sauryGβ, cutlassfishGβ and bluefishGβ, respectively. The lattice transformations described above confirmed that the space group and unit-cell parameters of the fish-derived Gβ crystals were essentially identical to those of the salmon-derived crystals (Wagner *et al.*, 2024 ▸) (Table 2 ▸). In the fish-derived Gβ crystals analyzed in this study, guanines exist as keto-N7H tautomers, which is also the case in salmon-derived crystals [Fig. 4 ▸(*a*)]. Crystal packing similarity among the Gβ crystal forms was evaluated using the Crystal Packing Similarity tool in *Mercury* (Macrae *et al.*, 2020 ▸) by com­paring clusters con­taining 30 symmetry-related mol­ecules after optimal superposition. Across all analyzed samples, the r.m.s. deviation (RMSD) values ranged from 0.021 to 0.113 Å (Table 3 ▸). Among the fish-derived guanine crystals, the RMSD values ranged from 0.021 to 0.087 Å, indicating no significant deviations in crystal packing (green coloured data in Table 3 ▸). These findings conclusively establish that the fish-derived crystals in this study con­tain the pre­viously reported Gβ polymorph.

### MicroED analysis of fish-derived Gα crystals

For the fish-derived Gα crystals (sauryGα, cutlassfishGα and bluefishGα), reprocessing with a predefined unit-cell parameter set of (3.6, 9.8, 16.5, 90, 96, 90) resulted in 42, 26 and 4 data­sets, respectively. Among these, the 37, 23 and 4 highest-resolution data­sets were selected for final merging (blue coloured data in Table 2 ▸). Structural analysis achieved a com­pleteness exceeding 90% and resolutions of 0.57 and 0.55 Å for saury and cutlassfish, respectively. In the case of bluefish, a com­pleteness of 73.0% was obtained, with a resolution of 0.69 Å. Data col­lection for bluefish was conducted at two different camera lengths: 400 (maximum resolution: 0.55 Å) for 1273 crystals and 500 mm (maximum resolution: 0.69 Å) for 854 crystals. BluefishGα crystals were identified exclusively in data­sets acquired at a 500 mm camera length, indicating that the observed 0.69 Å resolution was constrained by the instrumental resolution limit rather than by crystal quality. The initial structure, assigned to *P*2_1_/*c*, was solved and refined, yielding *R*_1_ values of 9.63, 11.07 and 6.43% for sauryGα, cutlassfishGα and bluefishGα, respectively.

Our analysis revealed that the space group and unit-cell parameters of the fish-derived Gα crystals closely matched those of the synGα crystals (Tables 1 ▸ and 2 ▸). In the fish-derived Gα crystals analyzed in this study, guanine mol­ecules exist as keto-N7H tautomers, as in the synGα crystals [Fig. 4 ▸(*b*)]. Crystal packing similarity among the Gα crystal forms was further evaluated using the Crystal Packing Similarity tool in *Mercury*. Across all analyzed samples, the RMSD values ranged from 0.024 to 0.064 Å (Table 4 ▸). Among the fish-derived guanine crystals, the RMSD values ranged from 0.024 to 0.039 Å, confirming no significant deviations in the crystal packing (blue coloured data in Table 4 ▸). These findings conclusively establish that the fish-derived crystals in this study con­tain the pre­viously reported Gα polymorph.

### Structural com­parison of guanine mol­ecules in fish-derived crystals

To evaluate the degree of structural conservation among the fish-derived guanine crystals, we conducted a com­parative analysis of guanine mol­ecular structures obtained from salmon-, Pacific saury-, Pacific cutlassfish- and blue damselfish-derived crystals, as well as synthetic guanine crystals (Table 5 ▸). Across all analyzed samples, the RMSD values ranged from 0.0054 to 0.0458 Å. Among the fish-derived guanine crystals, the RMSD values ranged from 0.0054 to 0.0288 Å, with β-polymorph crystals exhibiting RMSD values ranging from 0.0054 to 0.0288 Å (green coloured data in Table 5 ▸), while the α-polymorph crystals displayed RMSD values ranging from 0.0069 to 0.0128 Å (blue coloured data in Table 5 ▸). These results indicate no significant structural deviations among the analyzed samples. Thus, despite substantial differences in taxonomy, habitat and ecological adaptation among the fish species examined, the same guanine polymorphs (anhydrous β- and α-guanine) are con­sis­tently employed in structural colouration.

## Discussion

In this study, we determined the crystal structure of guanine in fish species exhibiting both constant and variable structural colour (Fig. 4 ▸). Previous MicroED research established that salmon exclusively incorporate guanine crystals as Gβ (Wagner *et al.*, 2024 ▸). However, our MicroED analysis reveals that the fish species examined in this study also employ guanine crystals as Gα [Fig. 4 ▸(*b*)]. This finding is con­sis­tent with prior PXRD studies, which identified Gα as a minor com­ponent (Hirsch *et al.*, 2015 ▸; Pinsk *et al.*, 2022 ▸). A fundamental limitation of MicroED is its inability to provide bulk sample information. The discrepancy between previous MicroED research and prior PXRD studies is likely attributable to the number of crystals analyzed. Our findings highlight the necessity of high-throughput MicroED data col­lection and also underscore the benefit of integrating MicroED with PXRD.

Our results demonstrate the utility of our semi-automated MicroED data col­lection and processing system for enabling large-scale structural analysis. For synthetic guanine crystals, the hit rate – defined as the ratio of successfully reprocessed data­sets to the total number of collected data­sets – was 11.2% at pH 2 and 13.0% at pH 10 (Table 1 ▸). For fish species exhibiting constant structural colour, the hit rate was com­parable or lower, at 14.0% for Pacific saury and 5.4% for Pacific cutlassfish. However, for fish species with variable structural colour, such as blue damselfish, the hit rate was further reduced to 0.85% (Table 2 ▸). The primary factor limiting the hit rate is likely the difficulty of sample preparation rather than the data-col­lection or processing workflow itself. During the extraction of guanine crystals from fish tissues, a substantial amount of biological contamination is often co-extracted, including muscle fragments, melanin-con­taining particles and other cellular debris. These contaminants can sometimes be difficult to distinguish from guanine crystals during crystal selection and automated data col­lection, resulting in the acquisition of data­sets that do not originate from the target crystals. Without automation, manually collecting and pro­cessing hundreds to thousands of data­sets would be impractical. This workflow may also be applicable to other biogenic crystalline materials that are inherently small or structurally heterogeneous, con­tain minor polymorphic com­ponents or are associated with substantial biological contamination that com­plicates con­ven­tional single-crystal analysis. The ability to collect and merge dif­frac­tion data from large numbers of submicrometer-sized crystals could facilitate structural studies of a wide range of biologically derived crystalline systems, including biogenic crystals found on spider surfaces, in scallop eyes and in chameleon skin (Gur *et al.*, 2017 ▸; Wagner *et al.*, 2024 ▸).

**Figure 5:**
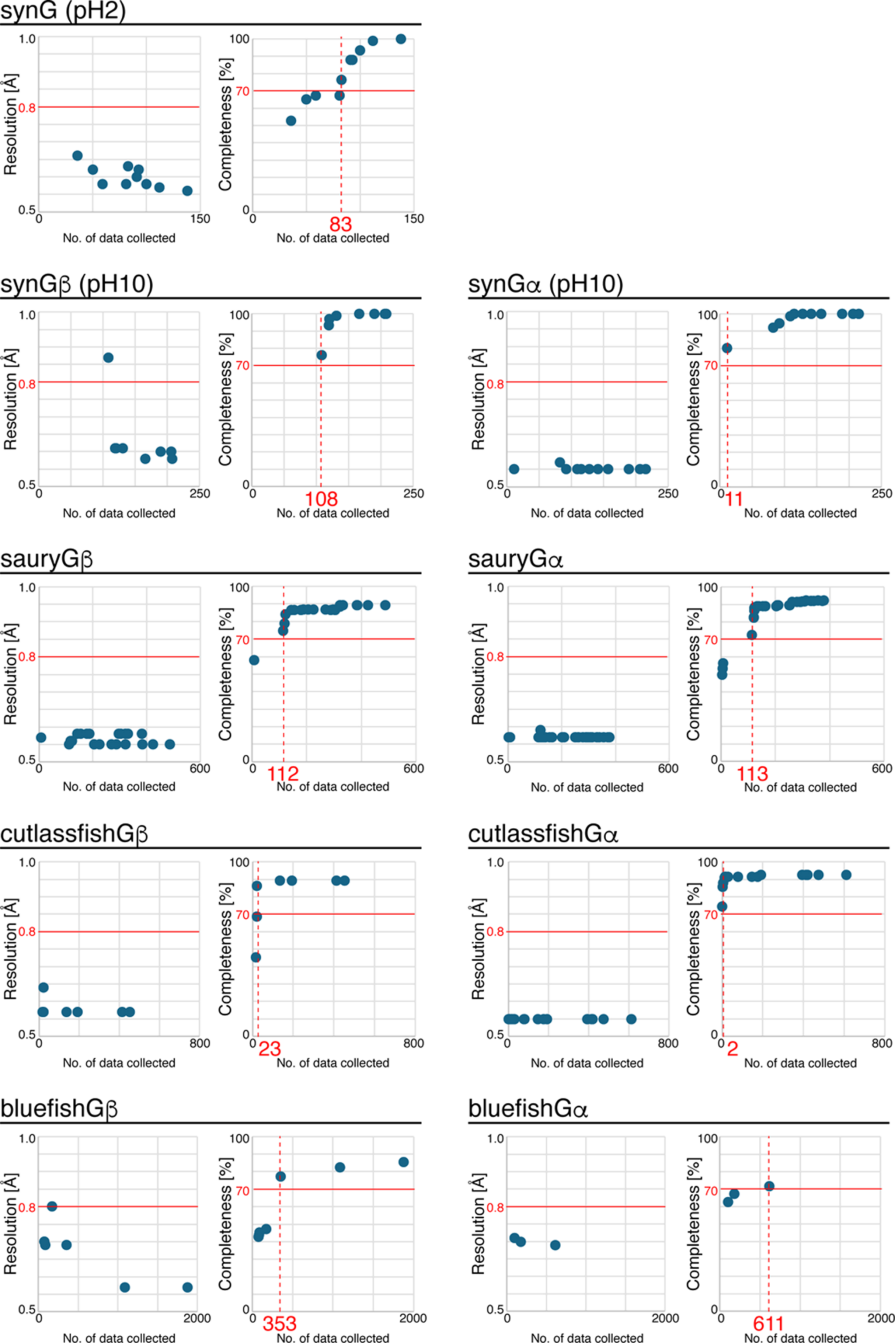
Relationship between the number of collected data­sets and resolution and com­pleteness in MicroED analysis of synthetic and fish-derived guanine crystals. The *x* axis represents the number of collected data­sets. Each point represents the com­pleteness or resolution obtained after cumulatively merging all successfully processed data­sets available at that stage of data col­lection. Red numbers and dashed lines indicate the number of data­sets required to reach 70% com­pleteness.

We further explored the correlation between the number of collected data­sets and both resolution and com­pleteness in the MicroED analysis of the synthetic and fish-derived guanine crystals (Fig. 5 ▸). Each point represents the com­pleteness or resolution obtained after cumulatively merging all successfully processed data­sets available at that stage of data col­lection. We first assessed the correlation between data­set count and resolution (left panels for each sample in Fig. 5 ▸). As high-resolution dif­frac­tion data are often desirable for accurate small-mol­ecule structure determination and analysis, achiev­ing sub-0.8 Å resolution is considered practically important. The plots showed that, with the exception of synGβ, all conditions achieved resolutions better than 0.8 Å from the initial data­sets used for scaling, highlighting the high crystal quality of fish-derived guanine crystals. It is notable that, in some cases, additional data­sets may have substanti­ally lower dif­frac­tion quality than those already included in the merged data­set. When such lower-quality data­sets are incorporated to increase com­pleteness, the overall resolution limit of the merged data­set may decrease. This trade-off explains why some plots show a decrease in resolution as more data­sets are included in the analysis. Next, we examined the correlation between data­set count and com­pleteness (right panels for each sample in Fig. 5 ▸). Given that 70% com­pleteness is typically adequate for initial structure determination of guanine crystals, surpassing this threshold is of practical importance. As mentioned above, the primary factor limiting the accumulation of mergeable data­sets is likely the difficulty of sample preparation rather than the data-col­lection or processing workflow itself. In the case of synGβ, sauryGβ, sauryGα, bluefishGβ and bluefishGα, more than 100 data­sets had to be collected before a sufficient number of successfully processed data­sets became available for merging to achieve 70% com­pleteness. In contrast, synGα, cutlassfishGβ and cutlassfishGα exceeded 70% com­pleteness using only 11, 23 and 2 data­sets, respectively. This reflects the specific characteristics of these samples, including biological contamination, preferred orientation and substantial crystal-to-crystal variability. It is noteworthy that curled or bent carbon support films may provide an effective means of mitigating preferred-orientation effects, particularly for sheet-like crystals such as biogenic guanine, and may facilitate the col­lection of more com­plete dif­frac­tion data­sets (Wennmacher *et al.*, 2019 ▸).

In this study, we determined the tautomeric forms of guanine in all the crystal structures analyzed. In synG, guanine adopts the keto-N9H tautomer, whereas in all other crystal forms (synGβ/α, sauryGβ/α, cutlassfishGβ/α and bluefishGβ/α), guanine adopts the keto-N7H tautomer. The H-atom positions defining these two tautomeric forms are directly sup­ported by Fourier difference maps calculated from the MicroED data (Fig. S2). In synG, the N9-bound H atom participates in inter­molecular guanine–guanine hy­dro­gen-bonding inter­actions through N9_1_—H9_1_⋯N3_2_ and N3_1_⋯H9_2_—N9_2_ contacts, generating pairs of guanine mol­ecules. Together with O6_1_⋯H2*A*_3_—N2_3_ and N7_1_⋯H1_3_—N1_3_ inter­actions, these hy­dro­gen bonds assemble into a hexa­gonal arrangement of guanine mol­ecules surrounding water mol­ecules. In contrast, in the Gβ and Gα crystal forms, the N7-bound H atom participates in inter­molecular guanine–guanine hy­dro­gen-bonding inter­actions through O6_1_⋯H7_2_—N7_2_ and N7_1_—H7_1_⋯O6_2_ contacts. These inter­actions constitute one of the two major guanine–guanine hy­dro­gen-bonding networks present in the Gβ and Gα crystals; the second network involves O6_1_⋯H2*B*_3_—N2_3_, N1_1_—H1_1_⋯N3_3_ and N2_1_—H2*A*_1_⋯N9_3_ inter­actions. In both tautomeric forms, the H atom defining the tautomer participates directly in inter­molecular hy­dro­gen-bonding inter­actions, indicating that tautomerism plays an important role in determining the hy­dro­gen-bonding network and crystal packing of guanine.

The presence of polymorphs in fish-derived guanine crystals provides novel insights into biomineralization processes. Our results suggest that these fish may possess an intrinsic ability to form both Gα and Gβ within their iridophore cells. The biological significance of these polymorphic variants remains unclear at this stage. If these structural polymorphs exhibit distinct optical properties, it would be particularly com­pelling to investigate how their functionalities are differentially exploited. Additionally, the mechanisms underlying the formation, intra­cellular transport and spatial organization of these polymorphs within iridophore cells remain open questions that warrant further investigation. To gain deeper insights into the natural arrangement and functional roles of guanine crystals in their biological context, future studies should integrate *in situ* methodologies, such as focused ion beam scanning electron microscopy combined with MicroED (*in situ* FIB-SEM-MicroED). These approaches will be essential for understanding how the anhydrous α- and β-polymorphs contribute to structural colouration and optical functionality within iridophore cells.

A potential concern regarding our findings relates to the temporal stability of guanine polymorphs. The PXRD study of synthetic guanine indicated that synGβ crystals can gradually transform into synGα over time (Gur *et al.*, 2016 ▸). Therefore, although prior PXRD studies identified synGα as a minor com­ponent, they considered it a possible transformation product of synGβ during purification (Hirsch *et al.*, 2015 ▸; Pinsk *et al.*, 2022 ▸). However, although the thermostability of synGα is estimated to be higher than that of synGβ, the calculated difference in stability between the two is minimal. Indeed, a previous study also mentioned that both polymorphs may appear in biogenic crystals (Hirsch *et al.*, 2015 ▸). To rigorously evaluate whether the fish-derived Gα arises as a transformation product, data­sets should be collected immediately after fish euthanasia. In this case, direct MicroED analysis of intact iridophore tissues remains challenging because crystal-con­taining cells and the surrounding biological matrix often require substantial thinning or crystal isolation before electron dif­frac­tion analysis can be performed. PXRD would be a more appropriate technique for such an investigation. An alternative approach would be to evaluate whether the sample-preparation procedure itself influences the observed polymorphic distribution. A rigorous assessment of such effects would require a pure synGβ starting material that could be subjected to the same extraction workflow used for fish-derived crystals, followed by evaluation of possible polymorphic conversion to synGα using PXRD or MicroED. Such analyses would help distinguish intrinsic biological regulation from potential sample-preparation effects. However, no established method is currently available for stably preparing pure synGβ crystals. Consequently, this control ex­peri­ment was not feasible within the scope of the present study, although we consider it an important direction for future investigation.

Another unresolved issue is the precise mol­ecular com­position of so-called ‘guanine crystals’. Several studies suggest that biogenic crystals may incorporate additional mol­ecular com­ponents, potentially influencing their optical and structural properties (Jordan *et al.*, 2012 ▸; Pinsk *et al.*, 2022 ▸; Wagner *et al.*, 2024 ▸). Addressing this issue will require a broader analysis encom­passing a diverse array of crystal samples from different fish species and distinct anatomical regions. The semi-automated MicroED data col­lection and processing system developed in this study provides a powerful platform for systematically investigating such com­positional variations. Large-scale automated analysis will be instrumental in identifying potential structural and com­positional heterogeneity within biogenic guanine crystals.

## Supplementary Material

Crystal structure: contains datablock(s) 2527795_bluefishga, 2531576_bluefishgb, 2531578_sauryga, 2531579_saurygb, 2531582_cutlassfishga, 2531584_cutlassfishgb, 2531593_syng_ph2, 2531601_synga_ph10, 2531603_syngb_ph10, global. DOI: 10.1107/S2053229626006236/yd3070sup1.cif

Structure factors: contains datablock(s) 2527795_bluefishga. DOI: 10.1107/S2053229626006236/yd30702527795_bluefishgasup2.hkl

Structure factors: contains datablock(s) 2531576_bluefishgb. DOI: 10.1107/S2053229626006236/yd30702531576_bluefishgbsup3.hkl

Structure factors: contains datablock(s) 2531578_sauryga. DOI: 10.1107/S2053229626006236/yd30702531578_saurygasup4.hkl

Structure factors: contains datablock(s) 2531579_saurygb. DOI: 10.1107/S2053229626006236/yd30702531579_saurygbsup5.hkl

Structure factors: contains datablock(s) 2531582_cutlassfishga. DOI: 10.1107/S2053229626006236/yd30702531582_cutlassfishgasup6.hkl

Structure factors: contains datablock(s) 2531584_cutlassfishgb. DOI: 10.1107/S2053229626006236/yd30702531584_cutlassfishgbsup7.hkl

Structure factors: contains datablock(s) 2531593_syng_ph2. DOI: 10.1107/S2053229626006236/yd30702531593_syng_ph2sup8.hkl

Structure factors: contains datablock(s) 2531601_synga_ph10. DOI: 10.1107/S2053229626006236/yd30702531601_synga_ph10sup9.hkl

Structure factors: contains datablock(s) 2531603_syngb_ph10. DOI: 10.1107/S2053229626006236/yd30702531603_syngb_ph10sup10.hkl

Supporting information file. DOI: 10.1107/S2053229626006236/yd30702531593_syng_ph2sup11.cml

Additional figures and tables. DOI: 10.1107/S2053229626006236/yd3070sup12.pdf

CCDC references: 2531603, 2531601, 2531593, 2531584, 2531582, 2531579, 2531578, 2531576, 2527795

## Figures and Tables

**Table 1:** Comparison of data col­lection and analysis of synthetic guanine crystals Orange, green and blue coloured data indicate synG, synGβ and synGα, respectively.

	Guanine monohydrate	SynG pH 2	Guanine anhydrous β	Guanine anhydrous α	SynGβ pH 10	SynGα pH 10
Method	SC-XRD	MicroED	PXRD	SC-XRD	MicroED	
Temperature (K)	298	92	295	120	92	
No. of data­sets collected	–	143	–	–	216	
No. of data­sets processed	–	48	–	–	35	
No. of data­sets reprocessed	–	16	–	–	9	19
(with unit-cell parameters)		(3.6, 11, 16.5,			(3.6, 8.8, 18.5,	(3.6, 9.8, 16.5,
		90, 96, 90)			90, 90, 83)	90, 96, 90)
Reprocessed/collected (%)	–	11.2	–	–	4.2	8.8
No. of data­sets used for scaling	–	14	–	–	9	13
Resolution (Å)	–	0.56	–	0.90	0.58	0.55
Completeness (%)	–	100	–	99.4	100	100
*R*_1_*	–	0.1600	–	0.0587	0.1415	0.1204
Space group						
Initially obtained	*P*2_1_/*n*	*P*2_1_/*n*	*P*2_1_/*c*	*P*2_1_/*c*	*P*2_1_/*n*	*P*2_1_/*c*
Transformed					*P*2_1_/*c*	
Unit-cell parameters						
*a* (Å)	16.510 (8)	3.6227 (5)	3.6317 (1)	3.5530 (16)	3.6369 (10)	3.6114 (8)
*b* (Å)	11.277 (8)	11.3187 (14)	18.4214 (11)	9.693 (4)	18.674 (4)	9.8783 (13)
*c* (Å)	3.645 (5)	16.651 (4)	9.8138 (10)	16.345 (7)	9.963	16.654 (3)
β (°)	96.8 (1)	96.087 (17)	117.945 (4)	95.748 (6)	118.46	95.69 (2)
Polymorph	monohydrate	monohydrate	anhydrous β	anhydrous α	anhydrous β	anhydrous α
Reference	Thewalt *et al.* (1971 ▸)	This study	Wagner *et al.* (2024 ▸)	Guille *et al.* (2006 ▸)	This study	

Notes: (*) *R*_1_ = Σ||*F*_o_| − |*F*_c_|| / Σ|*F*_o_| for reflections with *F*_o_ > 4σ(*F*_o_).

**Table 2:** Comparison of data col­lection and analysis of fish-derived guanine crystals Green and blue coloring indicate fish-derived Gβ and Gα, respectively.

	Salmon	SauryGβ	SauryGα	CutlassfishGβ	CutlassfishGα	BluefishGβ	BluefishGα
Method	MicroED	MicroED	MicroED	MicroED	MicroED	MicroED	MicroED
Temperature (K)	293	92					
No. of data­sets collected	3	506		651		2127	
No. of data­sets processed	–	63		34		21	
No. of data­sets reprocessed	–	29	42	9	26	14	4
(with unit-cell parameters)		(3.6, 8.8, 18.5,	(3.6, 9.8, 16.5,	(3.6, 8.8, 18.5,	(3.6, 9.8, 16.5,	(3.6, 8.8, 18.5,	(3.6, 9.8, 16.5,
		90, 90, 83)	90, 96, 90)	90, 90, 83)	90, 96, 90)	90, 90, 83)	90, 96, 90)
Reprocessed/collected (%)	–	5.7	8.3	1.4	4.0	0.66	0.19
No. of data­sets used for scaling	–	23	37	8	23	9	4
Resolution (Å)	0.67	0.55	0.57	0.57	0.55	0.57	0.69
Completeness (%)	87.4	89.5	92.7	89.8	92.9	85.7	73.0
*R*_1_*	0.195	0.0946	0.0963	0.0944	0.1107	0.0778	0.0643
Space group							
Initially obtained	*P*2_1_/*n*	*P*2_1_/*n*	*P*2_1_/*c*	*P*2_1_/*n*	*P*2_1_/*c*	*P*2_1_/*n*	*P*2_1_/*c*
Transformed	*P*2_1_/*c*	*P*2_1_/*c*	–	*P*2_1_/*c*	–	*P*2_1_/*c*	–
Unit-cell parameters							
*a* (Å)	3.630 (8)	3.6008 (9)	3.5953 (7)	3.6089 (15)	3.5993 (6)	3.626 (2)	3.608 (8)
*b* (Å)	18.34 (4)	18.5647 (18)	9.8020 (7)	18.531 (3)	9.8370 (6)	18.567 (4)	9.832 (4)
*c* (Å)	9.803 (19)	9.8988	16.5701 (15)	9.9156	16.5211 (13)	9.9373	16.467 (11)
β (°)	117.94 (6)	118.30	95.818 (13)	118.16	95.656 (11)	117.93	95.89 (14)
Polymorph	anhydrous β	anhydrous β	anhydrous α	anhydrous β	anhydrous α	anhydrous β	anhydrous α
Reference	Wagner *et al.* (2024 ▸)	This study					

Notes: (**) *R*_1_ = Σ||*F*_o_| − |*F*_c_|| / Σ|*F*_o_| for reflections with *F*_o_ > 4σ(*F*_o_).

**Table 3:** Crystal packing similarity in Gβ of fish-derived and synthetic guanine crystals (Å) Green coloured data indicates fish-derived Gβ.

	SynGβ	SalmonGβ	SauryGβ	CutlassfishGβ	BluefishGβ
SynGβ	–	–	–	–	–
SalmonGβ	30/30	–	–	–	–
	0.113				
SauryGβ	30/30	30/30	–	–	–
	0.058	0.080			
CutlassfishGβ	30/30	30/30	30/30	–	–
	0.056	0.077	0.021		
BluefishGβ	30/30	30/30	30/30	30/30	–
	0.046	0.087	0.046	0.031	

**Table 4:** Crystal packing similarity in Gα of fish-derived and synthetic guanine crystals (Å) Blue coloured data indicates fish-derived Gα.

	SynGα	SauryGα	CutlassfishGα	BluefishGα
SynGα	–	–	–	–
SauryGα	30/30	–	–	–
	0.054			
CutlassfishGα	30/30	30/30	–	–
	0.049	0.026		
BluefishGα	30/30	30/30	30/30	–
	0.064	0.039	0.024	

**Table 5:** RMSD of guanine mol­ecules in fish-derived and synthetic guanine crystals (Å) RMSD was calculated without H atoms. For cells con­taining two values, the lower value was obtained after inversion. Green and blue coloured data indicate fish-derived Gβ and Gα, respectively.

	Syn			Salmon	Saury		Cutlassfish		Bluefish	
	G	Gβ	Gα	Gβ	Gβ	Gα	Gβ	Gα	Gβ	Gα
SynG	–	–	–	–	–	–	–	–	–	–
SynGβ	0.0378	–	–	–	–	–	–	–	–	–
	0.0378									
SynGα	0.0403	0.0225	–	–	–	–	–	–	–	–
	0.0384	0.0151								
SalmonGβ	0.0458	0.0392	0.0372	–	–	–	–	–	–	–
	0.0412	0.0374	0.0372							
SauryGβ	0.0348	0.0166	0.0255	0.0274	–	–	–	–	–	–
	0.0348	0.0166	0.0184	0.0239						
SauryGα	0.0361	0.0250	0.0140	0.0263	0.0199	–	–	–	–	–
	0.0334	0.0196	0.0140	0.0263	0.0113					
CutlassfishGβ	0.0380	0.0186	0.0265	0.0272	0.0054	0.0211	–	–	–	–
	0.0380	0.0186	0.0196	0.0248	0.0054	0.0132				
CutlassfishGα	0.0400	0.0246	0.0151	0.0258	0.0189	0.0069	0.0189	–	–	–
	0.0373	0.0187	0.0151	0.0258	0.0095	0.0069	0.0095			
BluefishGβ	0.0410	0.0207	0.0171	0.0288	0.0183	0.0138	0.0181	0.0103	–	–
	0.0380	0.0160	0.0171	0.0288	0.0075	0.0138	0.0061	0.0103		
BluefishGα	0.0414	0.0288	0.0214	0.0246	0.0213	0.0128	0.0213	0.0103	0.0156	–
	0.0369	0.0243	0.0214	0.0246	0.0144	0.0128	0.0145	0.0103	0.0156	

## Data Availability

The crystal structure data described in this article have been deposited with the CCDC. The deposition numbers are as follows: synthetic guanine monohydrate, pH2 (synG), CCDC-2531593; synthetic guanine anhydrous β, pH10 (synGβ), CCDC-2531603; synthetic guanine anhydrous α, pH10 (synGα), CCDC-2531601; Pacific saury anhydrous β (sauryGβ), CCDC-2531579; Pacific saury anhydrous α (sauryGα), CCDC-2531578; Pacific cutlassfish anhydrous β (cutlassfishGβ), CCDC-2531584; Pacific cutlassfish anhydrous α (cutlassfishGα), CCDC-2531582; blue damselfish anhydrous β (bluefishGβ), CCDC-2531576; blue damselfish anhydrous α (bluefishGα), CCDC-2527795. All data-processing scripts used in this study are available *via* GitHub at https://github.com/Tsukuba-MicroED/data_process.
