# Supplementary material for: Semi-automated MicroED system unveils multiple polymorphs in fish-derived guanine crystals
Source: Acta Crystallogr C Struct Chem. 2026 Jun 22;82(Pt 7):310–23. doi: 10.1107/S2053229626006236 (PMC13330800; doi:10.1107/S2053229626006236)
Supplement: Supplementary file 12 [file c-82-00310-sup12.pdf]

STRUCTURAL CHEMISTRY

Volume 82 (2026)

Supporting information for article:

Semi-automated MicroED system unveils multiple polymorphs in fish-derived guanine crystals

Minato Nakazawa, Seishu Hayashi, Keita Tanaka, Kenji Iwasaki, Makoto Goda, Yusuke Yamada and Naruhiko Adachi

Semi-automated MicroED system unveils multiple polymorphs in fish-derived guanine crystals

Minato Nakazawa,[a] Seishu Hayashi,[a] Keita Tanaka,[a] Kenji Iwasaki,[a] Makoto Goda,[b]* Yusuke Yamada[c]* and Naruhiko Adachi[a]*

[a] Life Science Center for Survival Dynamics, Tsukuba Advanced Research Alliance (TARA), University of Tsukuba, 1-1-1 Tennodai, Tsukuba, Ibaraki 305-8577, Japan

[b] Institute of Photonics Medicine, Hamamatsu University School of Medicine, 1-20-1 Handayama, Chuo-ku, Hamamatsu, Shizuoka, 431-3192, Japan

[c] International Center for Synchrotron Radiation Innovation Smart (SRIS), Tohoku University, 468-1 Aramaki aza aoba, Aoba-ku, Sendai, Miyagi 980-0845, Japan

*Correspondence e-mails: cyano@muh.biglobe.ne.jp, yusuke.yamada.a6@tohoku.ac.jp, adachi.naruhiko.fu@u.tsukuba.ac.jp

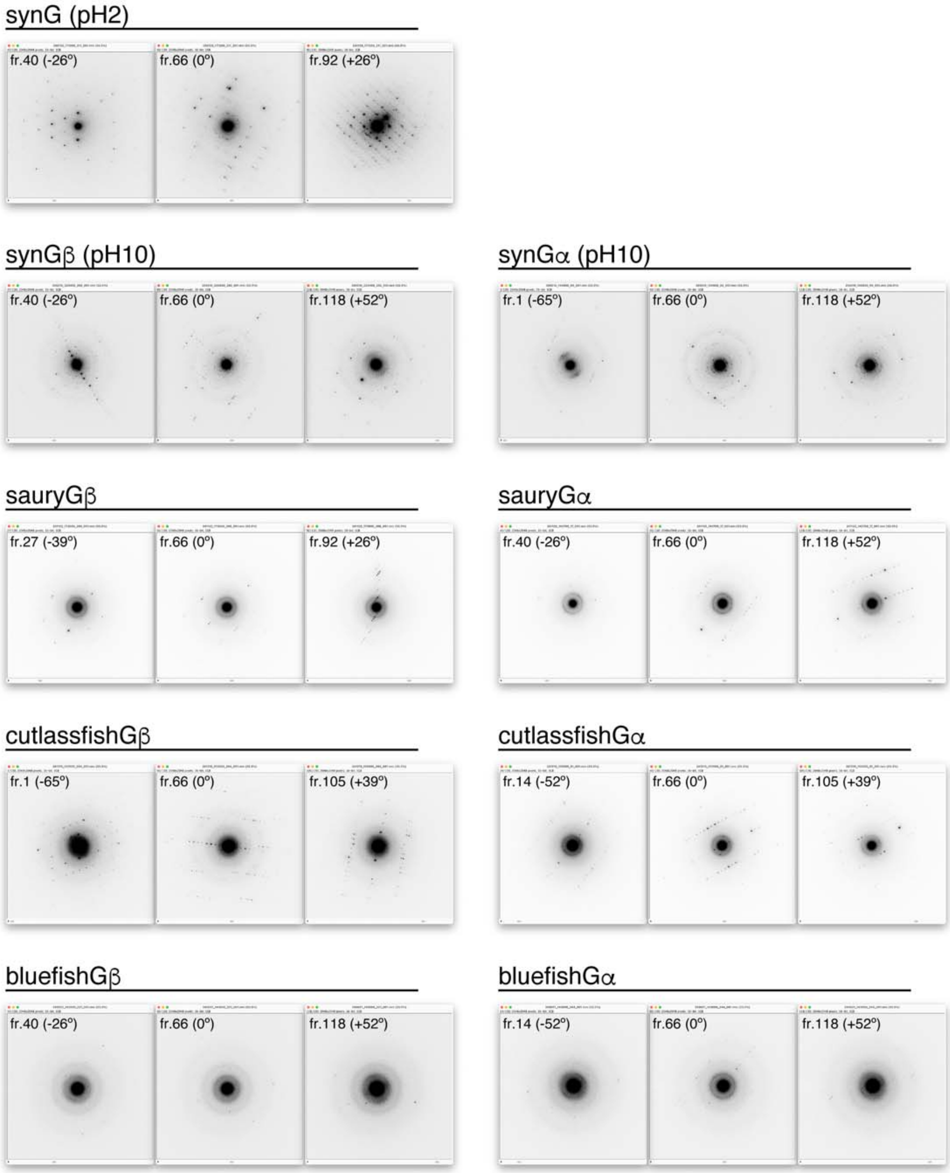

Figure S1 Representative diffraction frames of synthetic and fish-derived guanine crystals. Frame numbers and stage tilt angles are shown in the upper-left corner of each panel. Imperfect eucentric alignment can cause crystals to drift partially out of the illuminated area during continuous rotation, and the grid bar may obstruct the electron beam at certain tilt angles. For each dataset, the first and last frames in which diffraction spots were clearly observed are shown. These examples illustrate factors affecting the effective angular range and completeness of the diffraction data. The diffraction patterns extend to approximately 0.55 Å resolution at the detector edge, except for bluefishGα and bluefishGβ, which extend to approximately 0.69 Å resolution.

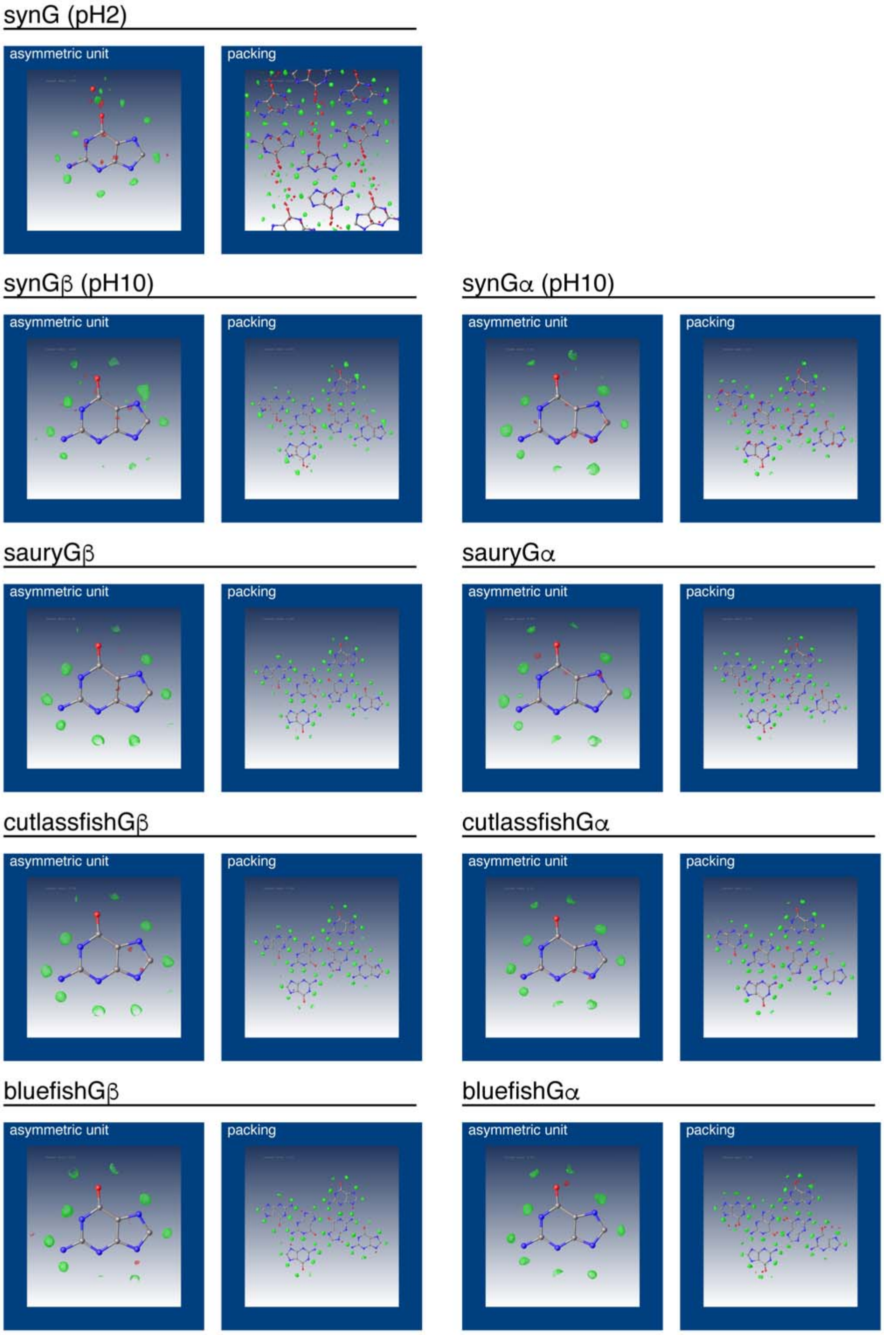

Figure S2 Omit map of synthetic and fish-derived crystals. After refinement, hydrogen atoms were deleted, and Fo-Fc maps were calculated. In each sample, the left panel shows the model (N: blue; O: red; C: grey) and map (positive: green; negative: red) of the guanine molecules in the asymmetric unit, and the right panel shows the corresponding packing arrangement.

Table S1 Data collection and refinement statistics of synthetic guanine crystals.

	SynG, pH2	SynGβ, pH10	SynGα, pH10
CCDC No.	2531593	2531603	2531601
Formula	C5H5N5O1-H2O	C5H5N5O1	C5H5N5O1
Data collection			
Microscope	CRYO ARM200	CRYO ARM200	CRYO ARM200
Voltage [kV]	200	200	200
Wavelength [Å]	0.02508	0.02508	0.02508
Detector	Rio 16M	Rio 16M	Rio 16M
Software	SerialEM	SerialEM	SerialEM
Temp [K]	92	92	92
Camera length [mm]	400 (408)*	400 (408)*	400 (408)*
Data analysis			
Processing	DIALS	DIALS	DIALS
Resolution [Å]	0.56	0.58	0.55
No. of datasets used for scaling	14	9	13
No. of total reflections	87,374	45,435	91,418
No. of unique reflections	4,055	3,201	3,726
Completeness [%]	100	100	100
Solve	SHELXT	SHELXT	SHELXT
Refinement	SHELXL	SHELXL	SHELXL
R_1**	0.1600	0.1415	0.1204
R_{int}	0.3166	0.3847	0.2469
wR_2	0.4276	0.4035	0.3444
Goodness-of fit	1.164	1.060	1.171
Shift	0.010	0.000	0.001
Extinction coefficient	99038(9)	30539(11)	44672(7)
Space group	$P2_1/n$	$P2_1/n$	$P2_1/c$
Unit-cell parameter			
a [Å]	3.6227(5)	3.6369(10)	3.6114(8)
b [Å]	11.3187(14)	18.674(4)	9.8783(13)
c [Å]	16.651(4)	8.829(2)	16.654(3)
β [º]	96.087(17)	97.23(3)	95.69(2)
Volume	678.9(2)	594.9(3)	591.20(19)
Z	4	4	4
Z’	1	1	1

*Parentheses show a calibrated camera length [mm].

**$R_1 = \Sigma||Fo| - |Fc|| / \Sigma|Fo|$ for reflections with $Fo > 4\sigma(Fo)$.

Table S2 Data collection and refinement statistics of fish-derived crystals.

	sauryGβ	sauryGα	cutlassfishGβ	cutlassfishGα	bluefishGβ	bluefishGα
CCDC No.	2531579	2531578	2531584	2531582	2531576	2527795
Formula	C5H5N5O1	C5H5N5O1	C5H5N5O1	C5H5N5O1	C5H5N5O1	C5H5N5O1
Data collection						
Microscope	CRYO ARM200	CRYO ARM200	CRYO ARM200	CRYO ARM200	CRYO ARM200	CRYO ARM200
Voltage [kV]	200	200	200	200	200	200
Wavelength [Å]	0.02508	0.02508	0.02508	0.02508	0.02508	0.02508
Detector	Rio 16M	Rio 16M	Rio 16M	Rio 16M	Rio 16M	Rio 16M
Software	SerialEM	SerialEM	SerialEM	SerialEM	SerialEM	SerialEM
Temp [K]	92	92	92	92	92	92
Camera length [mm]	400 (408)*	400 (408)*	400 (408)*	400 (408)*	400, 500 (408, 509)*	400, 500 (408, 509)*
Data analysis						
Processing	DIALS	DIALS	DIALS	DIALS	DIALS	DIALS
Resolution [Å]	0.55	0.57	0.57	0.55	0.57	0.69
No. of datasets used for scaling	23	37	8	23	9	4
No. of total reflections	168,149	253,001	49,499	197,828	33,102	9,570
No. of unique reflections	3,298	3,058	2,973	3,425	2,869	1,334
Completeness [%]	89.5	92.7	89.8	92.9	85.7	73.0
Solve	SHELXT	SHELXT	SHELXT	SHELXT	SHELXT	SHELXT
Refinement	SHELXL	SHELXL	SHELXL	SHELXL	SHELXL	SHELXL
R_1**	0.0946	0.0963	0.0944	0.1107	0.0778	0.0643
R_{int}	0.2261	0.2864	0.1561	0.2569	0.1513	0.1280
wR_2	0.3198	0.3028	0.3159	0.3274	0.2251	0.1625
Goodness-of fit	1.119	1.192	1.060	1.167	0.947	0.793
Shift	0.005	0.002	0.008	0.008	0.003	0.000
Extinction coefficient	64541(4)	62707(6)	42936(4)	99509(5)	7573(6)	1796(18)
Space group	$P2_1/n$	$P2_1/c$	$P2_1/n$	$P2_1/c$	$P2_1/n$	$P2_1/c$
Unit-cell parameter						
a [Å]	3.6008(9)	3.5953(7)	3.6089(15)	3.5993(6)	3.626(2)	3.608(8)
b [Å]	18.5647(18)	9.8020(7)	18.531(3)	9.8370(6)	18.567(4)	9.832(4)
c [Å]	8.7835(9)	16.5701(15)	8.8074(17)	16.5211(13)	8.840(3)	16.467(11)
β [º]	97.147(17)	95.818(13)	96.98(3)	95.656(11)	96.68(5)	95.89(14)
Volume	582.60(17)	580.94(13)	584.6(3)	582.10(11)	591.1(4)	581.1(14)
Z	4	4	4	4	4	4
Z’	1	1	1	1	1	1

*Parentheses show a calibrated camera length [mm].

**$R_1 = \Sigma||Fo| - |Fc|| / \Sigma|Fo|$ for reflections with $Fo > 4\sigma(Fo)$.
